# Profiles of Childhood Trauma in Women With Substance Use Disorders and Comorbid Posttraumatic Stress Disorders

**DOI:** 10.3389/fpsyt.2019.00674

**Published:** 2019-10-18

**Authors:** Annett Lotzin, Johanna Grundmann, Philipp Hiller, Silke Pawils, Ingo Schäfer

**Affiliations:** ^1^Department of Psychiatry and Psychotherapy, University Medical Center Hamburg-Eppendorf, Hamburg, Germany; ^2^Center for Interdisciplinary Addiction Research, University of Hamburg, Hamburg, Germany; ^3^Department of Medical Psychology, University Medical Center Hamburg-Eppendorf, Hamburg, Germany

**Keywords:** addiction, alcohol, comorbidity, women, caregiving, abuse, neglect

## Abstract

**Background:** It is increasingly becoming accepted that substance use disorders, including substance abuse and substance dependence, are closely related to childhood trauma and posttraumatic stress disorders. Among women with substance use disorders, the majority report sexual, physical or emotional abuse, or neglect. However, it is poorly understood which types of childhood trauma co-occur in women with substance use disorders and how combinations of different types and severities of childhood trauma are related to clinical characteristics. This information is important to inform treatment of substance use disorders.

**Aim:** The first aim of this research was to investigate profiles of childhood trauma in female patients with substance use disorders and posttraumatic stress disorders. The second aim was to examine relationships between these childhood trauma profiles and addiction characteristics or current clinical symptoms.

**Methods:** We includeda 343 treatment-seeking women with substance use disorders and comorbid posttraumatic stress disorders according to DSM-IV. Five types of childhood trauma (sexual abuse, physical abuse, emotional abuse) were measured using the Childhood Trauma Questionnaire. Addiction characteristics were assessed by using the Addiction Severity Index-lite. Current severity of clinical symptoms was determined by the Symptom-Checklist-27. Latent profile analysis was conducted to distinguish profiles of childhood trauma. Analysis of variance was applied to examine the relationship between childhood trauma profiles and addiction characteristics or severity of clinical symptoms.

**Results:** Nine out of ten women reported at least one type of childhood abuse or neglect. Four different childhood trauma profiles could be distinguished that characterized different types and severities of childhood trauma: ‘Low trauma’; ‘Moderate sexual abuse and emotional abuse’; ‘Severe sexual abuse and emotional abuse’; and ‘Severe levels of all types of trauma’. Profiles with more severe levels of childhood trauma showed an earlier age at initiation and escalation of substance use. Furthermore, childhood trauma profiles were related to current severity of depressive symptoms, dysthymic symptoms, sociophobic symptoms, and distrust.

**Conclusion:** In women with substance use disorders and posttraumatic stress disorders, childhood trauma profiles can inform about addiction characteristics and severity of a wide range of clinical symptoms. This information is essential to understand current treatment needs and should be systematically assessed in women with substance use disorders and trauma exposure.

## Introduction

Childhood trauma, including sexual and physical abuse, but also emotional abuse, emotional neglect and physical neglect (1), is a risk factor of developing mental disorders. Approximately 30% of mental disorders are accounted for exposure to childhood trauma (2). Substance use disorders (SUD) are among the most frequent mental disorders following traumatic events. SUD occur when the use of a drug (e.g., alcohol, cannabis or cocaine) leads to clinically significant impairment, including health problems, social withdrawal, and failure to meet major responsibilities at work, school or home (3). SUD are more prevalent in men than in women. For example, seven out of hundred men develop an alcohol use disorder at some point in their life, but only one out of hundred women (4). As SUD are less common in women, women with SUD are generally understudied (5).

Among both men and women with SUD, childhood trauma is highly prevalent. In patients with alcohol use disorders, 22–74% report at least one type of childhood abuse or neglect (6, 7). Female patients more often report severe forms of childhood trauma, particularly sexual abuse (8).

A history of childhood trauma impacts on the development, severity and course of SUD. Patients with alcohol use disorders exposed to childhood trauma develop the disorder at an earlier age (9, 10) and show more severe alcohol abuse (9, 11) than patients without childhood trauma.

Posttraumatic stress disorder (PTSD) is another mental disorder that is closely related to childhood trauma. PTSD is characterized by intrusive memories and nightmares of the trauma, hypervigilance (indicating enhanced threat sensitivity), and avoidance of places, activities or things that could remind a person of the traumatic event (3). Individuals with both SUD and PTSD manifest more severe clinical symptoms and lower psychosocial functioning (12).

In contrast to SUD, PTSD is more common among women: Three out of hundred women develop PTSD at some point of their life, but only one out of hundred men (4). After exposure to a potentially traumatic event, women are more likely to develop PTSD than men (13, 14). This finding might be explained by biological sex differences, but also by the fact that women are more often exposed to severe forms of interpersonal trauma, particularly sexual abuse (15, 16).

Among patients with alcohol dependence, women show about twice as high rates of a current PTSD (26–27%) than men (14–24%) (17, 18). Similar relationships between gender and SUD have been found for other substances than alcohol. Women with opioid use disorder, cocaine use disorder, cannabis use disorder or sedative use disorder showed twice as high prevalence rates of a current PTSD (50–53%) compared to men (14–32%) (19, 20).

Given that childhood trauma and PTSD are closely related to SUD (21), particularly in women, trauma exposure should be systematically assessed in this patient group. Thereby, the co-occurrence of different types and severities of childhood trauma should be considered, as most patients with trauma exposure report multiple types of events (6). The systematic assessment of these profiles of childhood trauma in women with SUD may inform about current mental health problems and related treatment needs. However, no study has identified profiles of childhood trauma in women with SUD and trauma exposure, or has examined how these profiles are related to current health-related outcomes.

So far, childhood trauma profiles have been predominantly examined in male patients with SUD (22–24). Among patients with alcohol dependence, six childhood trauma profiles could be distinguished that comprised different types and severities of trauma (22). The patients’ trauma profiles were differently associated with current severity of addiction-related problems in the domains of drug use, psychiatric symptoms, family relationships and social relationships. These results in male patients with alcohol dependence indicated that profiles of childhood trauma may better inform about current severity of addiction-related problems than the common distinction between trauma exposure versus no trauma exposure. Among male patients with SUD or polysubstance abuse, five childhood trauma profiles could be distinguished that were related to psychiatric problems (24). Tubman et al. (25) distinguished three profiles of childhood trauma in adolescents with SUD that were associated with severity of current psychiatric symptoms.

Although childhood trauma in SUD is closely related to PTSD, profiles of childhood trauma have rarely been examined in patients with SUD and comorbid PTSD. One study (26) assessed three different types of childhood trauma (psychological maltreatment, physical abuse, and sexual abuse) in a sample of trauma exposed clinic-referred adolescents. The authors assigned the participants to three different trauma groups, according to different combinations of the measured trauma types. Adolescents with both psychological maltreatment and physical abuse showed greater PTSD symptoms than the remaining groups.

In summary, childhood trauma profiles have been identified in different SUD patient groups that were associated with important mental health outcomes in all of the studies that have been conducted so far. These studies exclusively or predominantly included male patients, and PTSD comorbidity was not assessed. In women with SUD, childhood trauma profiles have not been examined so far. Furthermore, among patients with SUD and comorbid PTSD, childhood trauma profiles are unknown for both male and female patients.

Therefore, the first aim of this research was to investigate profiles of childhood trauma in women with SUD and PTSD. The second aim was to examine the relationships between these childhood trauma profiles and addiction characteristics or current clinical symptoms. We hypothesized that childhood trauma profiles with a greater number and/or severity of childhood trauma types would show unfavorable addiction characteristics and greater current clinical symptoms, compared to profiles with a lower number and severity of childhood trauma.

## Methods

### Design

The data of this study are derived from the baseline data of a larger intervention trial among patients with SUD and PTSD (27) (DRKS00004288). 

### Participants

Subjects were included in the study if they were (1) female, (2) aged between 18 and 65 years, (3) diagnosed with a substance abuse or substance dependence according to DSM-IV, (4) diagnosed with PTSD or subthreshold PTSD (i.e., criterion A, B, and either C or D) according to DSM-IV (28) and if they were (5) willing to participate in the study.

Subjects were excluded from study participation if they (1) were diagnosed with a psychosis according to DSM-IV (28), (2) showed severe cognitive impairment during screening or (3) reported intravenous drug use within four weeks before the start of study.

### Procedures

The participants of this trial were recruited in Germany between September 2012 and June 2015 in addiction or mental health inpatient and outpatient counseling and treatment facilities. The study was also promoted by advertisements in public venues. Written informed consent was obtained from all participants. Participants received €20 to compensate for their expenses. Data were collected from October 2012 to June 2015.

### Measures

Sociodemographic characteristics were obtained by a self-constructed interview. Psychiatric diagnoses of SUD (i.e., substance abuse and substance dependence) and PTSD were assessed according to DSM-IV criteria.

DSM-IV (28) defines a diagnosis of substance abuse as a maladaptive pattern of substance use leading to clinically significant impairment or distress, as manifested by one (or more) of the following, occurring within a 12-month period: (1) Recurrent substance use resulting in a failure to fulfil major role obligations at work, school, or home; (2) Recurrent substance use in situations in which it is physically hazardous; (3) Recurrent substance-related legal problems; (4) Continued substance use despite having persistent or recurrent social or interpersonal problems caused or exacerbated by the effects of the substance.

In contrast, a diagnosis of substance dependence is defined as a maladaptive pattern of substance use, leading to clinically significant impairment or distress, as manifested by three or more of the following criteria, occurring any time in a 12-month period: (1) Tolerance: (a) a need for markedly increased amounts of the substance to achieve intoxication or desired effect, or (b) markedly diminished effect with continued use of the same amount of the substance; (2) Withdrawal: (a) the characteristic withdrawal syndrome for the substance, or (b) the same substance is taken to relieve or avoid withdrawal symptoms; (3) The substance is often taken in larger amounts or over a longer period than intended; (4) There is a persistent desire or unsuccessful efforts to cut down or control substance use; (5) A great deal of time is spent in activities necessary to obtain the substance, use the substance, or recover from its effects; (6) Important social, occupational, or recreational activities are given up or reduced because of substance use; (7) The substance use is continued despite knowledge of having a persistent physical or psychological problem that is likely to have been caused or exacerbated by the substance (28).

A diagnosis of PTSD according to DSM-IV (28) is defined as fulfilling the following criteria: Criterion A: The person has been exposed to a traumatic event in which both of the following have been present: (1) the person experienced, witnessed, or was confronted with an event or events that involved actual or threatened death or serious injury, or a threat to the physical integrity of self or others; (2) the person’s response involved intense fear, helplessness, or horror. Criterion B: The traumatic event is persistently re-experienced in one or more of the following ways: (1) recurrent and intrusive distressing recollections of the event, including images, thoughts, or perceptions; (2) recurrent distressing dreams of the event; (3) acting or feeling as if the traumatic event were recurring; (4) intense psychological distress at exposure to internal or external cues that symbolize or resemble an aspect of the traumatic event; (5) physiological reactivity on exposure to internal or external cues that symbolize or resemble an aspect of the traumatic event. Criterion C: Persistent avoidance of stimuli associated with the trauma and numbing of general responsiveness as indicated by three (or more) of the following: (1) efforts to avoid thoughts, feelings, or conversations associated with the trauma; (2) efforts to avoid activities, places, or people that arouse recollections of the trauma; (3) inability to recall an important aspect of the trauma; (4) markedly diminished interest or participation in significant activities; (5) feeling of detachment or estrangement from others; (6) restricted range of affect (e.g., unable to have loving feelings); (7) sense of a foreshortened future (e.g., does not expect to have a career, marriage, children, or a normal life span). Criterion D: Persistent symptoms of increased arousal, as indicated by two (or more) of the following: (1) difficulty falling or staying asleep; (2) irritability or outbursts of anger; (3) difficulty concentrating; (4) hypervigilance; (5) exaggerated startle response. The described symptoms must be present for at least one month and must cause clinically significant distress or impairment in social, occupational, or other important areas of functioning.

The Structured Clinical Interview for DSM-IV (SCID) (29) was used to assess diagnoses of SUD and PTSD. The SCID is an established interview to measure psychiatric disorders according to DSM-IV criteria. It has proven sufficient reliability and validity for establishing clinical diagnoses (30).

The severities of the different types of childhood trauma were assessed using the Childhood Trauma Questionnaire (CTQ) (31, 32). The CTQ is a widely used self-report measure that assesses five types of childhood trauma (emotional abuse, physical abuse, sexual abuse, emotional neglect and physical neglect) using 25 items. The frequency of each type of trauma is rated on a five 5-point Likert scale ranging from 1 = “never” to 5 = “very often.” A total severity score (ranging from 25 to 125) and scores for each of the five subscales (ranging from 5 to 25) can be calculated. Severity classifications can be derived for each of the five trauma types by cut-off scores (31): “none or minimal” (emotional abuse 5–8, physical abuse 5–7, sexual abuse 5, emotional neglect 5–9, physical neglect 5–7), “low to moderate” (emotional abuse 9–12, physical abuse 8–9, sexual abuse 6–7, emotional neglect 10–14, physical neglect 8–9), “moderate to severe” (emotional abuse 13–15, physical abuse 10–12, sexual abuse 8–12, emotional neglect 15–17, physical neglect 10–12), and “severe to extreme” (emotional abuse ≥16, physical abuse ≥13, sexual abuse ≥ 13, emotional neglect ≥18, physical neglect ≥13). In patients with SUD, the CTQ demonstrated good internal consistencies, factorial, convergent and discriminant validity (32, 33).

Addiction characteristics were assessed by using the Addiction Severity Index-lite (ASI-lite) (34), the short form of the ASI (35). The ASI-lite is a structured interview yielding composite scores for severity of alcohol use and drug use (i.e., substances other than alcohol), among other addiction-related problems, in the last 30 days. ASI-lite composite scores range between 0 = “no problem” to 1 = “extreme problem,” with higher values indicating greater severity. For the present study, the alcohol and drug severity scores were combined to one score by using the highest severity score out of both scores as an indicator of substance use severity. Age at initiation of substance use and age at escalation of substance use were used for this study as additional outcomes, which are also assessed by the ASI-lite. The ASI has shown evidence for its internal construct validity, reliability, concurrent validity and utility for a wide range of research applications (36). Studies on the convergent validity of the ASI-lite with the ASI suggested that the ASI-lite alcohol and drug composite scores showed sufficient agreement with the ASI alcohol and drug composite scores (37).

Clinical symptoms within the last seven days were assessed by the Symptom-Checklist-27 (SCL-27) (38). The SCL-27 is a 27-item questionnaire that measures six dimensions of psychopathological symptoms on six subscales (‘Depressive symptoms’, ‘Dysthymic symptoms’, ‘Vegetative symptoms’, ‘Agoraphobic symptoms’, ‘Sociophobic symptoms’, and ‘Distrust’). Each of these subscales are measured by four to six items. Subscale scores are derived by calculating the mean of the respective items. The items of the subscale ‘Depressive symptoms’ measure depressive symptoms, e.g., “feeling blue” or “thoughts of death or dying.” The items of the subscale ‘Dysthymic symptoms’ measure less severe cognitive aspects of depressive symptoms, e.g., “trouble concentrating.” The subscale ‘Vegetative symptoms’ assesses somatoform symptoms, e.g., “heart pounding or racing” or “a lump in throat.” ‘Agoraphobic symptoms’ captures criteria of agoraphobia, e.g., “feeling afraid in open spaces or on streets.” The items of the subscale ‘Social phobia’ focus on aspects of self-confidence, e.g., “feeling very self-conscious with others.” The subscale ‘Symptoms of mistrust’ covers symptoms of suspicion and distrustfulness towards others, e.g., “feeling that most people cannot be trusted.” The SCL-27 proved satisfying reliability and very good factorial validity (38, 39).

As a potential confounding variable, PTSD symptom severity was assessed using the Posttraumatic Diagnostic Scale (PDS) (40). The PDS is a 49-item questionnaire that allows to assess severity of PTSD symptoms according to DSM-IV. The scale yields a total severity score. The PDS has demonstrated good reliability and validity in patients with alcohol dependence and comorbid PTSD (41).

### Statistical Analyses

Latent profile analysis (LPA) (42) was conducted in MPlus version 7.3 to identify profiles of childhood trauma, based on the severities of the five CTQ subscale scores. Latent profile analysis (LPA; also referred to as continuous latent class analysis) is a person-centered statistical technique that allows to classify individuals to homogeneous latent classes (i.e., profiles), based on their responses to observed variables, e.g., severities of different types of childhood trauma. Model parameters were estimated using maximum likelihood estimates. The robust maximum likelihood estimator was used to include participants with missing data. Complete data were available for 94.5% of the participants. To avoid local maxima, 800 random sets of starting values were used in the first step, 40 random sets were set in the second step of optimization and 20 initial stage iterations were used.

The Bayesian Information Criterion (BIC) (43), the sample size adjusted Bayesian information criterion (ssBIC) (44), the Lo-Mendell-Rubin adjusted likelihood ratio test (LMR test) (45) and the Bootstrapped Likelihood Ratio test (BLR test) (46) were used as model fit criteria to compare models with different class solutions. The information statistics BIC and ssBIC were used to compare the goodness-of-fit of competing models; the model with the lowest BIC is preferred. The BIC is based on the likelihood function and includes a penalty term for the number of parameters in the model to avoid over-fitting. The LMR and BLR test were used to compare models with increasing numbers of latent classes. In the LMR and BLR test, the estimated model fit is compared to a model with one class less. A *p*-value smaller than 0.05 suggests that the estimated model provides a better fit than the model with one class less (47). Entropy (48) was considered in the selection process as a measure of the accuracy of class assignment. Values greater than 0.80 indicate an acceptable probability of correct class assignment (49). The optimal number of classes was selected using model fit indicators, the interpretability of each class and parsimony. Other considerations included successful convergence, high entropy (greater than 0.80), no less than 1% of total count in a class, high posterior probabilities (near 1.0) and high proportions for the latent classes (all above 1%).

Effects of the childhood trauma classes on addiction-related characteristics, including age at initiation of substance use, age at escalation of substance use and current addiction severity, were analyzed by linear models. Age at initiation of substance use and age at escalation of substance use were analyzed in one multivariate analysis of variance (MANOVA) to account for multiple correlated outcomes. Wilks’ lambda was used as a multivariate F-test. Current addiction severity was analyzed in a separate ANOVA, because n = 71 participants had to be excluded from this analysis, because these participants were in inpatient addiction or psychiatric treatment at the time of assessment where substance use was prohibited, which would have biased the results. Effects of the childhood trauma classes on clinical symptoms, including depressive, dysthymic, vegetative, agoraphobic, sociophobic, and distrust symptoms, were analyzed in a second MANOVA. In all linear models, participants’ age, years of education and PTSD symptom severity were included as covariates to control for potential confounding, as these variables have been shown to be related to the outcomes in previous studies.

All variance inflation factors of the used variables were smaller than four, indicating low problems with multicollinearity (50). (M)ANOVA analyses were conducted using SPSS IBM Statistics Version 24 (IBMCorp., Armonk, NY).

## Results

### Sample Characteristics

N = 343 treatment-seeking women with SUD and (at least subsyndromal) PTSD were included in the study. Participants were 40.9 years (SD = 11.4) old, on average. Years of education ranged from 7 to 13 years, with a median of 10 years. Most women were born in Germany (n = 310, 90.4%). Eight out of ten women were unmarried (n = 287, 83.7%), eight out of ten were unemployed (n = 267, 77.8%). Five out of ten women received a monthly income of lower than €1000 (n = 186, 54.4%).

Nine out of ten women (n = 324, 94.5%) were diagnosed with a substance dependence, the remaining women (n = 19, 5.5%) were diagnosed with a substance abuse. Eight out of ten women (n = 290, 84.5%) were diagnosed with an alcohol use disorder. Five out of ten women (n = 165, 48.5%) fulfilled the diagnostic criteria for a cannabis use disorder, three out of ten women (n = 106, 31.2%) fulfilled the criteria for a sedative use disorder. Three out of ten women (n = 97, 28.5%) were diagnosed with a cocaine use disorder; three out of ten women (n = 96, 28.2%) were diagnosed with a stimulant use disorder other than cocaine; and two out of ten women (n = 73, 21.3%) were diagnosed with an opiate use disorder. Eight out of ten women (n = 270, 78.7%) consumed substances within the last 30 days. Six out of ten women (n = 226, 66.1%) previously participated in a substance abuse treatment.

Eight out of ten women (n = 248, 75.2%) fulfilled the criteria for comorbid PTSD; the remaining women fulfilled the criteria for subthreshold PTSD. Two out of ten women participated in prior trauma-related treatment (n = 80, 23.4%). Four out of ten women were diagnosed with a Major Depression (n = 153, 44.9%), six out of ten women were diagnosed with an anxiety disorder (n = 221, 64.4%). Six out of ten women attempted suicide at some point in their life (n = 197, 57.8%).

As defined by our inclusion criteria, all women were exposed to a traumatic event according to DSM-IV (28). Nine out of ten women (n = 320, 93.3%) reported at least one type of childhood abuse or neglect. Eight out of ten women reported at least moderate levels of emotional abuse (n = 267, 78.5%) or emotional neglect (n = 261, 76.5%); seven out of ten (n = 249, 72.8%) reported at least moderate sexual abuse, six out of ten (n = 209, 61.1%) reported at least moderate physical neglect; and five out of ten women (n = 179, 52.2%) reported physical abuse.

### Profiles of Childhood Trauma

The LMR test revealed a significant difference between the 4-class and 3-class solution (*p* = 0.026), but no significant difference between the 5-class and 4-class solution (*p* = 0.200, **Table 1**). However, the BLR remained significant for the 5-class solution (*p* < 0.001), indicating that the 5-class solution differed from the 4-class solution. The entropy values were greater than 0.80 for all models, indicating a good separation between the classes (48). The 4-class solution yielded meaningful profiles that included different severities of the five types of childhood trauma; the 5-class solution contained the same four childhood trauma profiles as the 4-class solution, and one additional class with a similar curve shape than the ‘Severe levels of all types of trauma’ class, but with higher mean severities of the different types of trauma. For the 4-class solution, the total counts per class were all higher than 1%, the posterior probabilities were near 1.0 and the proportions for the latent classes were all above 1%. On the basis of the fit indices and meaningful interpretability of the classes, the 4-class model was determined to best fit the data.

**Table 1:** Model fit indices of latent profiles of the severities of childhood traumatic events in female patients with substance use disorders and comorbid posttraumatic stress disorders (N = 343).

Class	*Log likelihood*	*BIC*	*ssBIC*	*LMR*	*LMR test p-value*	*BLR*	*BLR test p-value*	*Entropy*
1		10752.4	10720.7	–	–	–	–	–
2	-5097.7	10288.9	10238.1	-5347.0	.000	-5347.0	<.001	0.83
3	-5016.0	10160.4	10090.6	-5097.7	.056	-5097.7	<.001	0.82
4	-4974.3	10112.0	10023.2	-5016.0	.026	-5016.0	<.001	0.81
5	-4936.9	10072.3	9964.4	-4974.3	.200	-4974.3	<.001	0.84

BIC, Bayesian Information Criterion. ssBIC, sample size adjusted Bayesian Information Criterion. LMR, Lo-Mendell-Rubin adjusted likelihood ratio. BLR, Bootstrapped Likelihood Ratio.

**Figure 1:**
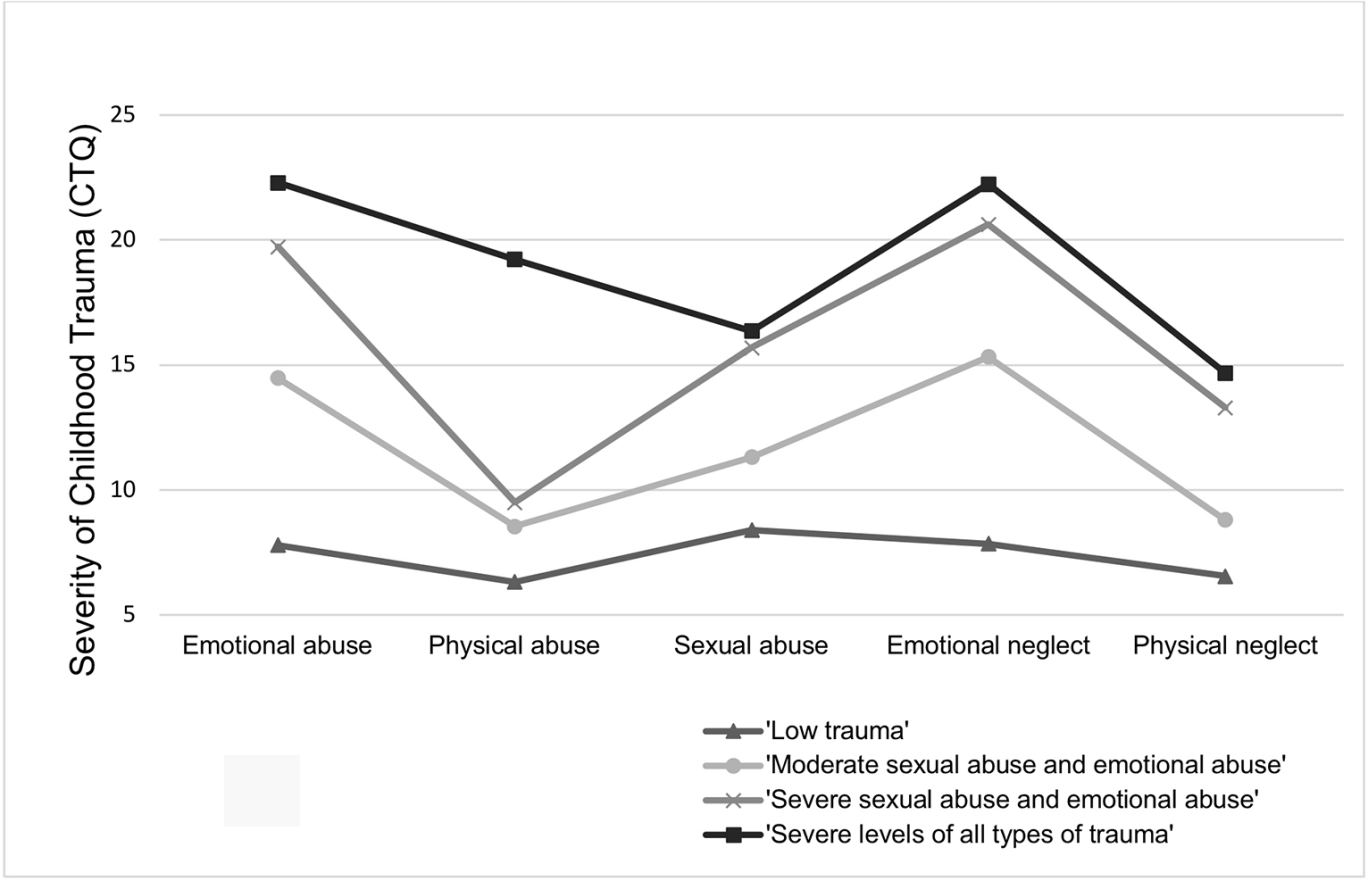
Profiles of childhood trauma in women with substance use disorders and posttraumatic stress disorders. CTQ, Childhood Trauma Questionnaire.

Profile 1 (n = 38, 11.1%) was representative of women with SUD and PTSD with minimal emotional and physical abuse, minimal emotional and physical neglect and low sexual abuse (**Table 2**, **Figure 1**). This profile was labelled ‘Low trauma’. Profile 2 (n = 114, 33.3%) characterized women with moderate sexual abuse, moderate emotional abuse and moderate emotional neglect, but low physical abuse and physical neglect, and was therefore labelled ‘Moderate sexual abuse and emotional abuse’. Profile 3 (n = 93, 27.1%) described women with severe sexual and emotional abuse, combined with severe emotional and physical neglect, but low physical abuse. This profile was labelled ‘Severe sexual abuse and emotional abuse’. Profile 4 (n = 97, 28.3%) clustered women with high severities of all types of childhood trauma, including severe emotional, physical and sexual abuse, as well as severe emotional and physical neglect, which was named ‘Severe levels of all types of trauma’.

**Table 2:** Clinical and sociodemographic characteristics of childhood trauma profiles (N = 343).

	Profile 1 ‘Low trauma’ (n = 38)	Profile 2 ‘Moderate sexual abuse and emotional abuse’ (n = 114)	Profile 3 ‘Severe sexual abuse and emotional abuse’ (n = 93)	Profile 4 ‘Severe levels of all types of trauma’ (n = 97)
	*M (SD)*	*M (SD)*	*M (SD)*	*M (SD)*
Age	39.66 (13.03)	39.89 (11.38)	40.62 (11.21)	42.98 (10.70)
Education (years)	10.71 (1.68)	10.90 (1.47)	10.68 (1.66)	10.38 (1.38)
Severity of childhood trauma				
Total severity	7.38 (2.98)	11.69 (3.58)	15.77 (3.73)	18.96 (3.94)
Emotional abuse	7.79 (2.86)	14.47 (3.54)	19.73 (2.78)	22.30 (2.60)
Physical abuse	6.32 (2.03)	8.55 (3.35)	9.51 (3.20)	19.23 (3.02)
Sexual abuse	8.39 (5.32)	11.31 (5.60)	15.69 (6.67)	16.36 (7.19)
Emotional neglect	7.84 (2.44)	15.32 (3.07)	20.63 (2.37)	22.24 (2.63)
Physical neglect	6.55 (2.25)	8.81 (2.34)	13.29 (3.65)	14.68 (4.24)
Age at initiation of SUD	19.53 (8.90)	17.02 (6.22)	16.79 (7.35)	17.81 (9.94)
Age at escalation of SUD	30.31 (13.19)	25.69 (10.2)	25.32 (11.53)	24.39 (11.28)
PTSD symptom severity	24.58 (10.97)	26.35 (9.75)	27.98 (9.36)	28.85 (9.27)
Severity of SUD	0.26 (0.28)	0.31 (0.29)	0.30 (0.28)	0.32 (0.28)
Severity of clinical symptoms				
Depressive symptoms	1.11 (0.90)	1.52 (0.93)	1.68 (0.86)	1.41 (0.95)
Dysthymic symptoms	1.43 (1.09)	1.70 (1.00)	1.95 (0.95)	1.62 (1.01)
Vegetative symptoms	1.19 (0.93)	1.19 (0.83)	1.36 (0.81)	1.39 (0.81)
Agoraphobic symptoms	0.89 (0.88)	1.07 (0.94)	1.19 (0.88)	1.16 (0.93)
Sociophobic symptoms	1.31 (1.04)	1.63 (1.05)	1.88 (0.99)	1.70 (1.05)
Distrust symptoms	0.96 (0.83)	1.40 (0.86)	1.53 (0.97)	1.58 (0.95)

SUD, Substance Use Disorder. PTSD, Posttraumatic Stress Disorder. Severity of childhood trauma was assessed by the Childhood Trauma Questionnaire. Addicted characteristics were measured with the Addiction Severity Index-lite. PTSD symptom severity was assessed by the Posttraumatic Diagnostic Scale. Clinical symptoms were measured using the Symptom-Checklist-27.

### Relations Between Profiles of Childhood Trauma and Addiction Characteristics

Women with a ‘Severe sexual abuse and emotional abuse’ profile reported an earlier initiation of substance use than women with a ‘Low trauma’ profile, after controlling for age, education and PTSD severity (**Table 3**). Women with a ‘Moderate sexual abuse and emotional abuse’ profile or a ‘Severe levels of all types of trauma’ profile reported an earlier initiation of substance use by trend, compared to women with the ‘Low trauma’ profile. Among the variables included in the model to control their effects on the outcomes (age, education, and PTSD symptom severity), age was significantly related to age at initiation of substance use, with older women showing later initiation of substance use.

**Table 3:** Effects of childhood trauma profiles on addiction characteristics (N = 343).

Variable	*b*	95% CI Lower	95% CI Upper	*p*	Partial η^2^
Age at initiation of substance use					
Profile 2 ‘Moderate sexual abuse and emotional abuse’^a^	-2.83	-5.83	0.17	.065	.012
Profile 3 ‘Severe sexual abuse and emotional abuse’^a^	-3.40	-6.48	-0.32	**.031**	**.016**
Profile 4 ‘Severe levels of all types of trauma’^a^	-2.82	-5.89	0.26	.072	.011
Years of education	0.06	-0.49	0.61	.827	.000
Age	0.27	0.19	0.34	**<.001**	**.142**
PTSD symptom severity	-0.05	-0.14	0.04	.279	.004
Age at escalation of substance use					
Profile 2 ‘Moderate sexual abuse and emotional abuse’^a^	-3.50	-7.30	0.30	.071	.011
Profile 3 ‘Severe sexual abuse and emotional abuse’^a^	-4.57	-8.47	-0.67	**.022**	**.018**
Profile 4 ‘Severe levels of all types of trauma’^a^	-6.39	-10.28	-2.50	**.001**	**.034**
Years of education	0.31	-0.38	1.01	.376	.003
Age	0.55	0.46	0.65	**<.001**	**.306**
PTSD symptom severity	0.00	-0.12	0.11	.952	.000
Severity of substance use disorder^b^					
Profile 2 ‘Moderate sexual abuse and emotional abuse’^a^	0.04	-0.07	0.16	.455	.002
Profile 3 ‘Severe sexual abuse and emotional abuse’^a^	0.03	-0.09	0.15	.627	.001
Profile 4 ‘Severe levels of all types of trauma’^a^	0.04	-0.08	0.16	.536	.001
Years of education	0.03	0.01	0.05	**.013**	**.023**
Age	0.01	0.00	0.01	**<.001**	**.048**
PTSD symptom severity	0.00	0.00	0.00	.874	.000

SUD, Substance Use Disorder. PTSD, Posttraumatic Stress Disorder. ^a^Reference category = Profile 1 ‘Low trauma’. Clinical symptoms were assessed by the Symptom-Checklist-27. ^b^n = 272, because 71 participants were in stationary addiction or psychiatric treatment where substance use was prohibited.

Age at escalation of substance use was also significantly affected by childhood trauma profiles (**Table 3**). Women with a ‘Severe sexual abuse and emotional abuse’ profile or a ‘Severe levels of all types of trauma’ profile reported earlier escalation of substance use, compared to women with a ‘Low trauma’ profile. Women with a ‘Moderate sexual abuse and emotional abuse’ profile showed an earlier escalation of substance use by trend, compared to women with a ‘Low trauma’ profile. Among the potential confounder variables, age was significantly related to escalation of substance use, with older women reporting later escalation of substance use.

Current severity of SUD was unrelated to childhood trauma profiles (**Table 3**). Among the confounding variables, age was significantly related to SUD severity, with older women reporting greater SUD severity. Education was also significantly positively related to current SUD severity, with more years of education being related to greater SUD severity.

### Relations Between Profiles of Childhood Trauma and Clinical Characteristics

A greater severity of depressive symptoms was shown by women with the ‘Moderate sexual abuse and emotional abuse’ profile and the ‘Severe sexual abuse and emotional abuse’ profile, compared to women with a ‘Low trauma’ profile, after controlling for age, education and PTSD severity (**Table 4**). Among the potential confounder variables (age, education, and PTSD symptom severity), education and PTSD symptom severity were significantly positively related to severity of depressive symptoms.

**Table 4:** Effects of childhood trauma profiles on current clinical symptom severity (N = 343).

Variable	*b*	95% CI Lower	95% CI Upper	*p*	Partial η^2^
Depressive symptoms					
Profile 2 ‘Moderate sexual abuse and emotional abuse’	0.31	0.00	0.62	**.049**	**.012**
Profile 3 ‘Severe sexual abuse and emotional abuse’^a^	0.44	0.12	0.76	**.007**	**.021**
Profile 4 ‘Severe levels of all types of trauma’^a^	0.16	-0.16	0.48	.336	.003
Years of education	0.08	0.02	0.14	**.008**	**.021**
Age	0.00	-0.01	0.01	.932	.000
PTSD symptom severity	0.04	0.03	0.05	**<.001**	**.162**
Dysthymic symptoms					
Profile 2 ‘Moderate sexual abuse and emotional abuse’	0.18	-0.16	0.51	.303	.003
Profile 3 ‘Severe sexual abuse and emotional abuse’^a^	0.37	0.03	0.72	**.034**	**.013**
Profile 4 ‘Severe levels of all types of trauma’^a^	0.03	-0.31	0.38	.847	.000
Years of education	0.08	0.02	0.14	**.013**	**.018**
Age	0.00	-0.01	0.01	.783	.000
PTSD symptom severity	0.05	0.04	0.06	**<.001**	**.192**
Vegetative symptoms					
Profile 2 ‘Moderate sexual abuse and emotional abuse’	-0.09	-0.37	0.19	.539	.001
Profile 3 ‘Severe sexual abuse and emotional abuse’^a^	0.02	-0.28	0.31	.913	.000
Profile 4 ‘Severe levels of all types of trauma’^a^	0.02	-0.27	0.32	.868	.000
Years of education	0.02	-0.04	0.07	.541	.001
Age	0.00	-0.01	0.01	.948	.000
PTSD symptom severity	0.04	0.03	0.05	**<.001**	**.188**
Agoraphobic symptoms					
Profile 2 ‘Moderate sexual abuse and emotional abuse’	0.08	-0.23	0.39	.594	.001
Profile 3 ‘Severe sexual abuse and emotional abuse’^a^	0.13	-0.19	0.45	.411	.002
Profile 4 ‘Severe levels of all types of trauma’^a^	0.07	-0.25	0.39	.672	.001
Years of education	0.00	-0.05	0.06	.906	.000
Age	0.00	-0.01	0.01	.915	.000
PTSD symptom severity	0.04	0.03	0.05	**<.001**	**.191**
Sociophobic symptoms					
Profile 2 ‘Moderate sexual abuse and emotional abuse’	0.29	-0.06	0.65	.108	.008
Profile 3 ‘Severe sexual abuse and emotional abuse’^a^	0.48	0.12	0.85	**.010**	**.020**
Profile 4 ‘Severe levels of all types of trauma’^a^	0.32	-0.04	0.69	.084	.009
Years of education	0.05	-0.02	0.11	.187	.005
Age	-0.01	-0.02	0.00	**.002**	**.027**
PTSD symptom severity	0.04	0.03	0.05	**<.001**	**.128**
Distrust symptoms					
Profile 2 ‘Moderate sexual abuse and emotional abuse’	0.36	0.03	0.68	**.031**	**.014**
Profile 3 ‘Severe sexual abuse and emotional abuse’^a^	0.45	0.12	0.78	**.008**	**.021**
Profile 4 ‘Severe levels of all types of trauma’^a^	0.48	0.15	0.81	**.005**	**.023**
Years of education	0.02	-0.05	0.08	.628	.001
Age	0.00	-0.01	0.01	.946	.000
PTSD symptom severity	0.03	0.02	0.04	**<.001**	**.108**

PTSD, Posttraumatic Stress Disorder. ^a^Reference category = Profile 1 ‘Low trauma’. Clinical symptoms were assessed by the Symptom-Checklist-27.

A greater severity of dysthymic symptoms were reported by women with a ‘Severe sexual abuse and emotional abuse’ profile, compared to women with a ‘Low trauma’ profile. Education and PTSD symptom severity were also significantly positively related to severity of dysthymic symptoms.

The childhood trauma profiles were not significantly related to severity of vegetative symptoms or agoraphobic symptoms. Instead, PTSD symptom severity was significantly positively related to severity of vegetative and agoraphobic symptoms.

More severe sociophobic symptoms were reported by women with a ‘Severe sexual abuse and emotional abuse’ profile, compared to women with a ‘Low trauma’ profile. Women with a ‘Severe levels of all types of trauma’ profile showed more severe sociophobic symptoms than women with a ‘Low trauma’ profile by trend. Age and PTSD symptom severity were significantly positively related to sociophobic symptom severity.

A greater severity of distrust symptoms were reported by women with a ‘Moderate sexual abuse and emotional abuse’ profile, a ‘Severe sexual abuse and emotional abuse’ profile, or a ‘Severe levels of all types of trauma’ profile, compared to women with a ‘Low trauma’ profile. PTSD symptom severity was significantly positively related to severity of distrust symptoms.

## Discussion

### Profiles of Childhood Trauma

In this study, we investigated profiles of childhood trauma among women with SUD and PTSD, an understudied population in SUD research. Nine out of ten women reported at least one type of childhood abuse or neglect. Remarkably, seven out of ten women reported moderate or severe childhood sexual abuse. The high prevalence of sexual abuse in women with SUD, relative to men with SUD, is consistent with earlier study results (21). Moderate or severe physical abuse was reported by five out of ten women. Compared to a predominantly male SUD sample (22), the prevalence of childhood physical abuse was lower in our female sample used in this study. This gender difference in the type of experienced trauma emphasizes the need of gender specific treatment programs. To date, these specific treatment needs among male and female patients with SUD are not appropriately addressed.

Among women with SUD and PTSD, we identified four distinct profiles of childhood trauma. Given that all participants of our study were diagnosed with at least subsyndromal PTSD, only one out of ten women belonged to a ‘Low trauma’ profile, characterized by low levels of interpersonal childhood trauma. The women assigned to this profile reported minimal levels of emotional and physical abuse, as well as minimal neglect, but low levels of sexual abuse. Hence, low levels of sexual abuse seem to take place in family environments that do not necessarily incorporate other types of childhood abuse or neglect.

Three out of ten women could be best described by a ‘Moderate sexual abuse and emotional abuse’ profile. These women had been exposed to moderate sexual abuse, combined with moderate emotional abuse and emotional neglect. In a study among predominantly male pathological gamblers (23), a comparable childhood trauma profile was identified, characterized by sexual abuse combined with emotional abuse and neglect. This profile comprised a particularly high rate of female patients. Among all female pathological gamblers that participated in this earlier study, four out of ten women belonged to this profile, which matches the proportion of women with SUD assigned to the ‘Moderate sexual abuse and emotional abuse’ profile in this study. Hence similar trauma profiles might exist for women with SUD across different types of addictive disorders.

Three out of ten women were grouped to a ‘Severe sexual abuse and emotional abuse’ profile, characterized by severe sexual and emotional abuse, combined with severe emotional and physical neglect. This profile was characterized by low physical abuse. In an earlier study among primarily male patients with SUD (22), a small subgroup of the sample was best described by a profile of severe sexual abuse, severe emotional neglect and moderate to severe emotional abuse, but no physical abuse. This profile was most often reported by women. In contrast, men that were exposed to severe levels of sexual abuse more often reported additional physical abuse. According to these results, sexual abuse seems to be more frequently combined with physical abuse in men than in women.

Thirty out of hundred women reported ‘Severe levels of all types of trauma’, comprising severe emotional, physical and sexual abuse, as well as severe emotional and severe physical neglect. This is a large proportion of the whole sample, compared to other research using addiction samples (22, 23). In an earlier study with primarily male patients with alcohol dependence (22), only four out of hundred patients belonged to this high-risk profile; in a study with predominantly male patients with gambling disorders (23), twenty out of hundred patients were assigned to a similar profile. The extremely high prevalence of this very severe trauma profile might be partly explained by the fact that this study only included women with SUD and at least subsyndromal PTSD. However, given that five out of ten women with SUD are affected by comorbid PTSD (19, 20), extreme levels of all types of trauma exposure seem to concern a large subgroup among the whole population of women with SUD.

Noteworthy, women of the ‘Severe sexual abuse and emotional abuse’ profile described more severe depressive and dysthymic symptoms compared to women of the ‘Severe levels of all types of trauma’ profile. The main difference between these two profiles was that the latter included additional severe physical abuse. One explanation of the lower levels of depressive and dysthymic symptoms in the ‘Severe levels of all types of trauma’ profile might be that women may respond to severe sexual and emotional abuse with internalizing symptoms, e.g., depression, whereas women might respond to severe physical abuse in addition to sexual abuse with externalizing symptoms, e.g., aggressive behavior (51) that may mask depressive symptoms. Consistent with this idea, women exposed to severe physical abuse in childhood had an increased risk to become a perpetrator by themselves in adulthood (52).

Comparing the profiles of childhood trauma identified in this study with the profiles found in earlier studies (22, 23), this analysis did not reveal a profile with moderate levels of emotional neglect, but no other types of childhood trauma. The absence of this profile can be explained by the fact that this study only selected women with a comorbid posttraumatic stress disorder, which is caused by extremely threatening active forms of traumatic events, such as physical and sexual abuse. A substantial amount of women with SUD but without PTSD might be characterized by an emotional neglect profile, which are underrepresented in this study. Future studies might include women with SUD but without PTSD in order to identify additional profiles of childhood trauma among women with SUD.

### Relations Between Profiles of Childhood Trauma and Addiction Characteristics

Childhood trauma profiles were related to addiction characteristics, after controlling for age, education and PTSD severity. The ‘Severe sexual abuse and emotional abuse’ profile significantly predicted earlier initiation of substance use, compared to a ‘Low trauma profile’. The ‘Moderate sexual abuse and emotional abuse’ and the ‘Severe levels of all types of trauma’ profile showed earlier initiation of substance use by trend. The women of these three trauma profiles initiated substance use three years earlier than the women of the ‘Low trauma’ profile, on average. These results concur with earlier research in SUD samples that showed that childhood trauma exposure was associated with earlier substance use (10). Similarly, more severe childhood trauma profiles were related to earlier onset of SUD in patients with alcohol dependence (22).

The ‘Severe sexual abuse and emotional abuse’ and the ‘Severe levels of all types of trauma’ profiles were significantly related to an earlier escalation of substance use, compared to a ‘Low childhood trauma’ profile. The ‘Moderate sexual abuse and emotional abuse’ profile was related to earlier escalation of substance use by trend. These findings are in agreement with earlier research that reported that a higher number and severity of childhood trauma was associated with earlier onset of substance abuse (9). Trauma exposure might be related to earlier initiation and escalation of substance use, as it may help to dampen negative cognitions and emotions during and after abuse, as well as to reduce intrusions and arousal related to PTSD. Consistent with this assumption, Runtz and Schallow (53) found that women with a sexual abuse exposure more often used dysfunctional forms of coping, such as alcohol use.

Childhood trauma profiles were unrelated to current severity of SUD. Although the mean severity of SUD was lower for the ‘Low trauma’ profile, there was substantial variation within this profile and the difference was not statistically significant, indicating that the mean effect of childhood trauma profiles on SUD severity was minimal. Previous studies that examined relations between SUD characteristics and childhood trauma (54, 55) or childhood trauma profiles (22) also reported no relations between these variables. As addiction severity is determined by multiple factors, other factors may have masked the effect of trauma on SUD severity. Alternatively, the missing association between trauma exposure and SUD severity might be due to methodological issues. Most of the studies mentioned above (22, 55) used the ASI or ASI-Lite to assess SUD severity, which might lack power to discriminate SUD severity between subgroups of patients with SUD. Although trauma exposure might be unrelated to SUD severity at intake, there is evidence that trauma exposure is related to lower reduction in SUD severity over course of SUD treatment (15), as well as to lower reduction in SUD severity after treatment (15, 54). Further studies should clarify the relationship between childhood trauma profiles and SUD severity using different measurement approaches than the ASI.

### Relations Between Profiles of Childhood Trauma and Clinical Characteristics

Childhood trauma profiles were related to severity of current clinical symptoms. The ‘Moderate sexual abuse and emotional abuse’ profile and the ‘Severe sexual abuse and emotional abuse’ profile were related to greater depressive symptoms, e.g., “feeling blue” or “thoughts of death or dying,” compared to a ‘Low trauma’ profile (**Table 4**). The ‘Severe sexual abuse and emotional abuse’ profile was also related to more severe dysthymic symptoms that indicated cognitive aspects of depressive symptoms, e.g., “trouble concentrating.” Relations between depressive symptoms and trauma exposure have been previously reported in SUD samples (54).

The ‘Severe sexual abuse and emotional abuse’ profile was related to more severe sociophobic symptoms, i.e., aspects of low self-confidence (39); the ‘Severe levels of all types of trauma’ profile was related to sociophobic symptoms by trend. These findings are consistent with the results of a previous study in which sexual abuse was related to greater social anxiety (56). A negative self-concept including low self-confidence is typically seen in individuals with complex PTSD (57).

All three profiles that included moderate to extreme interpersonal childhood abuse (‘Moderate sexual abuse and emotional abuse’, ‘Severe sexual abuse and emotional abuse’, ‘Severe levels of all types of trauma’) showed more severe symptoms of distrust. The highest levels of distrust were reported by women belonging to one of the two profiles including severe abuse (‘Severe sexual abuse and emotional abuse’ and ‘Severe levels of all types of trauma’). Symptoms of mistrust, i.e., suspicion and distrustfulness, characterize patients with complex PTSD (58).

When compared to a representative German population (38), all trauma profiles, including the ‘Low trauma profile’, characterized elevated levels of clinical symptoms. Nevertheless, type and severity of trauma exposure greatly varied within women with SUD and PTSD, pointing to the need to assess profiles of childhood trauma. Women with severe trauma profiles may need treatment that considers underlying vulnerabilities related to early chronic interpersonal trauma. This may include the modification of dysfunctional cognitive schemas that affect self-concept, mistrust, and interpersonal behavior.

### Secondary Results

Among the variables that were included in analysis to control their effect on dependent variables, we found that older women reported later initiation and escalation of substance use. Although most individuals with SUD initiate substance use in adolescence or adulthood, some women initiate substance use in mid or late adulthood (59). This earlier finding is reflected in our data. Older women also showed greater SUD severity than younger women. This result might be explained by the fact that age is correlated with lifetime alcohol consumption, which in turn is likely to be associated with greater SUD severity (60). As reported by a previous study (61), older women also showed less sociophobic symptoms, indicating that social anxiety might decline with age.

Women with more years of education showed greater SUD severity. This result is consistent with earlier results of a national German addiction survey (62) that found positive associations between socioeconomic status and SUD severity. More years of education were also associated with depressive symptoms and dysthymic symptoms, which might be related to greater SUD severity.

PTSD symptom severity was unrelated to addiction characteristics. This is an interesting result, given that it is generally assumed that substances are used to regulate PTSD symptoms, in terms of a self-medication hypothesis (63). In line with earlier research (64), PTSD symptom severity was associated with clinical symptoms other than SUD, including depressive, dysthymic, vegetative, agoraphobic, sociophobic, and distrust symptoms. These results indicate that women with SUD and PTSD suffer from complex comorbidities that should be addressed in treatment of women with SUD and PTSD.

### Strengths and Limitations

A strength of this study is that we examined a sample of women with SUD, an often overlooked and understudied patient group. We assessed a wide range of different childhood trauma types, including emotional abuse, emotional neglect, and physical neglect. These types of childhood trauma are often underrepresented in trauma research. However, we excluded male patients with SUD, which can be considered as a weakness of this study, as our findings cannot be generalized to men with SUD and PTSD. We were also unable to recruit a sample that was representative for the whole population of women with SUD and PTSD. However, our recruitment strategy resulted in a sample with various types and severities of SUD and childhood trauma.

We went beyond traditional approaches by investigating childhood trauma profiles including different severities and types of trauma. At the same time, we did not assess non-interpersonal types of childhood trauma. Future studies might assess these additional types of trauma and their relationship to addiction characteristics and clinical symptoms.

The cross-sectional design of this study does not allow conclusions about causal relationships between childhood trauma exposure, addiction characteristics and clinical symptoms. Due to the cross-sectional design, trauma exposure was assessed retrospectively, that might be related to recall bias. Another limitation of the study can be seen in the use of LPA that is an exploratory statistical technique to uncover latent homogeneous groups within a sample. Given its exploratory nature, no *a priori* assumptions about the number of classes were made (47). It is also worth mentioning that not all of the model fit statistics indicated that a 4-class solution best fitted the data. Further studies should examine whether a 4-class solution best describes childhood trauma profiles among women with SUD and PTSD.

DSM-IV criteria of SUD and PTSD were used to include participants in this study. The diagnostic criteria of DSM-5 significantly differ from the criteria outlined in DSM-IV. As a result, it is likely that we included participants in this study which might have not met the trauma criterion according to DSM-5 (65). Hence, the trauma profiles found in this study might diverge from profiles among women with SUD and PTSD diagnosed according to DSM-5.

Despite these limitations, the results of this study add important knowledge about childhood trauma profiles and their role for addiction characteristics and clinical symptoms in women with SUD and PTSD. Childhood sexual abuse was highly prevalent, and combinations of severe to extreme forms of childhood abuse and neglect were reported by the majority of women. Childhood trauma profiles explained early initiation and escalation of substance use, as well as a greater severity of a wide range of clinical symptoms. These findings have important implications for treatment of comorbid SUD and PTSD among women. Childhood trauma profiles should be routinely assessed to inform treatment. Given that childhood trauma profiles were related to a wide range of clinical symptoms beyond SUD and PTSD, these additional clinical symptoms should be also considered in treatment. As women with SUD are less adherent to treatment than men, treatment programs should address common reasons for lower treatment adherence by offering childcare and services specific for women’s issues (66). For example, mental health consequences of sexual abuse should be preferably treated in gender-specific groups (67).

## Conclusion

In a sample of women with SUD and PTSD, childhood trauma profiles with a greater severity and a higher number of childhood trauma were related to earlier initiation of substance use, earlier age at escalation of substance use and greater severity of a broad range of current clinical symptoms. According to these findings, childhood trauma profiles can provide a differentiated view about important addiction characteristics and current severity of a wide range of clinical symptoms. This information is essential to inform treatment needs in women with SUD and PTSD.

## Data Availability

The datasets generated for this study are not publicly available as the participants did not provide consent to make data available for the public.

## Ethics Statement

The study was approved by the local ethics committees of the medical associations of the study sites (Ethics Committee of the Medical Association Hamburg, Ethics Committee of the Hannover Medical School, Ethics Commission of the Medical Association of Westphalia-Lippe and the Medical Faculty of Westphalia Wilhelms University, Ethics Committee of the North Rhine-Westphalia Medical Association and Ethics Commission of the Medical Faculty of the University of Duisburg-Essen). The participants provided their written informed consent to participate in this study.

## Author Contributions

IS and SP designed the study. PH, JG and AL managed the conduct of the study. PH led the data assessment and data management. AL conducted the statistical analysis and wrote the first draft of the manuscript. AL, JG, PH, SP and IS contributed to and approved the final manuscript.

## Funding

This work was supported by the German Federal Ministry of Education and Research (BMBF) under grant number 01KR1203A.

## Conflict of Interest Statement

The authors declare that the research was conducted in the absence of any commercial or financial relationships that could be construed as a potential conflict of interest.
